# Women’s empowerment and fertility preferences in high fertility countries in Sub-Saharan Africa

**DOI:** 10.1186/s12905-019-0747-9

**Published:** 2019-04-05

**Authors:** Esso-Hanam Atake, Pitaloumani Gnakou Ali

**Affiliations:** 1 Department of Economics Sciences, University of Lome Togo, 01 BP 1515, Lome, Togo; 2 Department of Sociology, University of Lome (Togo), 01 BP 1515, Lome, Togo

**Keywords:** Fertility preferences, Empowerment, Francophone Sub-Saharan Africa, Women, Ideal number of children, Demographic and health surveys (DHS)

## Abstract

**Background:**

Nearly all countries with fertility levels of more than five children per woman are in Sub-Saharan Africa. Prestige, insurance in old age, and replacement in case of child deaths are related to preferences for large families. In this paper, we examine the association between women’s empowerment and fertility preferences of married women aged 35 years and above in four high fertility Francophone Sub-Saharan Africa (FSSA) countries, namely Burkina Faso, Mali, Niger and Chad.

**Method:**

The ideal number of children among married women and their ability to have the desired number of children are used to measure fertility preferences. We used principal component analysis to construct a multidimensional empowerment index. We then estimated negative binomial and logistic regression models to examine the association between women’s empowerment and fertility preferences. Data are from the most recent Demographic and Health Surveys (DHS) conducted in the countries included in the analysis.

**Results:**

Regardless of the country, more empowered women desire significantly fewer children compared with their less empowered counterparts. The first step to having fewer children is formulating programs to improve economic empowerment of women. The specific elements of women’s empowerment that were important for fertility preferences included education, skills development, decision-making power, and control over household resources. In addition, familial empowerment matters more than other dimensions of empowerment in influencing women’s ability to achieve the desired number of children in the FSSA countries included in the study.

**Conclusion:**

Paid employment and access to and control over resources are factors which, if improved upon, could significantly reduce the ideal number of children. By taking necessary steps, mass media can be used much more adequately to reduce ideal number of children in FSSA countries. In addition, the desire for many children could also be due to their participation in income-generating activities to improve the household’s socio-economic status. The findings suggests that improvement of women’s ability to have the desired number of children is a big challenge to which policy makers must pay careful attention.

## Background

Almost all the countries where the fertility rate exceeds five children per woman are in Sub Saharan Africa (SSA) [1]. While the total births per woman was estimated in 2016 at 4.8 in SSA, it was estimated at 3.3; 2.5; 2.1; and 1.6 respectively in the Arab world, South Asia, Latin America and Caribbean and the European Union [2]. Fertility trend shows that SSA is experiencing an extremely slow decline in fertility, compared to other regions of the world [3]. Fertility rates remain high, leading to high youth dependency [3]. Meanwhile, the proportion of women aged 15–49 who use a method of contraception and are married or in relationships does not exceed 22% in SSA, compared with 86% in East Asia and 72% in Latin America and the Caribbean [2]. In SSA, the use of modern contraceptives and other family planning strategies is minimal; which results in a high incidence of unintended and unwanted pregnancy [4, 5].

Women’s empowerment is identified as a key solution that can change the prevailing fertility and contraceptive use patterns in SSA [6–10]. The World Bank defines empowerment as “the process of enhancing an individual’s or group’s capacity to make purposive choices and to transform those choices into desired actions and outcomes” [11]. According to Kabeer [12], empowerment is “the expansion of people’s ability to make strategic life choices in a context where this ability was previously denied to them.” For others, empowerment is characterized by several components such as power to act, autonomy, participation, self-direction, self-determination, liberation, mobilization and self-confidence [13, 14]. Furthermore, higher educational attainment and increase in household wealth have positive effects on married women’s autonomy, including their decision-making power within the household [15].

There is a consensus that empowerment influences the reproductive health outcomes such as contraceptive use, fertility, and birth spacing. With regards to fertility preferences in SSA, studies reveal that greater household decision making was associated with a smaller ideal number of children [12]. In Guinea and Zambia, “women’s egalitarian gender-role attitudes were associated with a smaller ideal number of children” [9]. It was found that women who had no decision-making autonomy had 0.26 more children than women who had some autonomy in Zimbabwe [16]. In Eritrea, women’s ability to make decisions about daily household purchases is associated with the expression of a small ideal number of children and the desire to have no more children [17]. The relationship between empowerment and fertility preferences can also be analysed in the light of the demographic transition theory. Theories of demographic transitions consider female labour force participation one of the causes for actual declines in fertility and fertility preferences [12, 18]. Education plays an important role since most fertility-related behaviours change with women’s educational attainment: it influences age of getting married, competence in child health, and use of family planning [19].

A review of the literature shows that that generally, fertility preferences are proxied by desired family size, ideal number of children, and desire for additional children. However, especially in a socio-cultural context of high fertility, there could be another pathway through which women‘s empowerment affects fertility preferences. Empowerment may expand a woman’s agency and resources which enhance her ability to achieve her ideal number of children [20]. In this study, we explore whether a woman’s level of empowerment affects her ability to achieve her ideal number of children. Another important challenge is that, some studies found both positive and inverse associations between empowerment measures and fertility [9, 21, 22]. These results could be an artefact of the flaws in the measures of empowerment [23, 24]. There is consensus that empowerment is multidimensional and is expressed at multiple levels. However, there is contestation and debate regarding on which dimensions and levels matter most [10, 25]. In this study, we used a single empowerment index which takes into account all dimensions.

Another contribution of our study is that it focuses on poor and high fertility Francophone Sub-Saharan Africa (FSSA) countries (Burkina Faso, Chad, Mali and Niger). FSSA countries are the lowest ranked in the Human Development Index (HDI) of 2016, with, for example, Mali being ranked 175th, Burkina Faso 185th, Chad 186th, and Niger 187th out of 188 countries [26]. Fertility rate remains high in these countries where there is low access to family planning services: the average number of children per woman is 7.6 in Niger, 8.2 in Chad, 6 in Burkina Faso, and 6.1 in Mali [27–30]. The prevalence of contraceptive use is estimated at 8% in Niger, 5.4% in Chad, 15% in Burkina Faso and 10% in Mali [27–30]. Birth control remains a big challenge in FSSA countries. However, very little is known about the association between women’s empowerment and fertility preferences in the region.

FSSA countries are characterized by persistent unemployment and underemployment of women. For example, in Burkina Faso, participation in economic activity is higher among men than women [31]. In Mali, 51% of jobs are held by women and 49% held by their male counterparts [32]. However, only 3.6% of these 51% involve payment of salary or wages [32]. Moreover, early marriage is so prevalent in Mali that one out of two women marries before the age of 16.5, while the average age of men at marriage is 26.1 years [32]. Furthermore, the completion rate of junior secondary education was only 13% for girls in Niger, in 2015 [33]. In Chad, the number of girls is less than 10% of the overall number of students enrolled in school.

The aim of this study was to examine the association between women’s empowerment and fertility preferences in high fertility FSSA countries, controlling for socio-economic and demographic characteristics. It specifically answers the following research questions: is women’s empowerment associated with achieving the desired family size in FSSA? Which dimension of empowerment matters more in influencing women’s ability to achieve the desired number of children? We hypothesize that (i) an increase in women’s empowerment is associated with small family norms; (ii) economic empowerment matters more than other dimensions of empowerment in influencing women’s ability to achieve the desired number of children.

### Conceptual framework

**Fig. 1:**
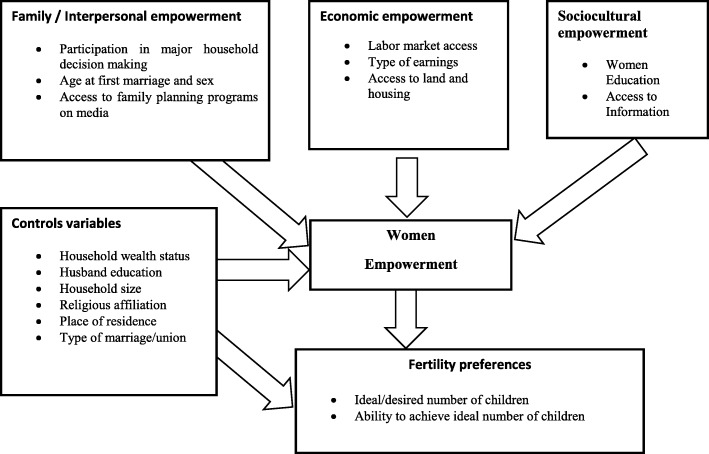
Empowerment Factors and Fertility preferences

The conceptual framework used to analyse the association between women’s empowerment and fertility preferences in FSSA countries is given in Fig. 1. According to the framework, there are three dimensions of a woman’s empowerment: socio-cultural dimension includes educational attainment and access to information, economic dimension entails access to paid work and ownership of house and land, and familial dimension which encompasses age and participation in all important household decisions [9, 25, 34]. Generally, a woman’s access to information, control of resources, and participation in decision-making change fertility preferences [35]. Schooling and exposure to media can help to empower women [35] and affect positively ideal family size [19]. Moreover, culture, religious beliefs, and gender relations play a critical role in household decisions about reproduction and hence overall fertility levels [6, 16, 36]. Furthermore, social norms, household wealth, spouse’s educational level and professional status, and place of residence affect fertility preferences [37].

## Methods

In this section, we describe the measurement of dependent variables and empowerment index, the analytic methods, and the source of data.

### Measurement of the dependent variable

A woman’s reproductive performance can be influenced by what is for her, the ideal number of children, and the ability to limit her fertility to that ideal number. Thus, from the perspective of empowerment, the ideal number of children married women desire and their ability to have just the desired number are the dependent variables.

The first dependent variable is ***the ideal number of children for each married woman***. The Demographic and Health Surveys (DHS) provide information on the ideal number of children for each married woman through the following question: “If you could go back to the time you did not have any children and could choose exactly the number of children to have in your whole life, how many would that be?”. The question is phrased slightly differently for those who do not have living children: “If you could choose exactly the number of children to have in your whole life, how many would that be?”. This question elicits both numerical and non-numerical responses. Whether the response is numerical or not, it has been taken into account in the analysis to minimize bias [9]. This strategy is consistent with those of previous studies which recommended the inclusion of non-numerical responses in the analysis of family size desires [9, 16, 38]. However, opinions are divided in the literature on the issue. Some studies show that those who give nonnumeric responses are likely to want more children and are less likely to adopt behaviours that lead to smaller families than those who provide numeric responses [9, 38]. Others studies found lack of a statistically significant difference in the indicators of empowerment for the two groups (numeric and nonnumeric) [9]. Preliminary findings from our study showed no significant differences in empowerment indicators between women who provided numerical responses and those who provided non-numerical ones (not shown). Thus, non-numerical responses were recorded to the mean value for the rest of the country sample [9].

The second dependent variable is ***the married woman’s ability to have just the desired number of children***. This variable is a dichotomous measure that provides information on the woman’s number of living children compared with the number of desired children. It is computed by subtracting the ideal number of children from the number of living children [9]. If the difference is greater than zero, the woman is considered as having more children than her stated ideal.

### Women’s empowerment index

In order to take into account the multidimensional nature of women’s empowerment, we use Principal Component Analysis (PCA) method to construct a single empowerment index [39]. The approach is informed by the different dimensions used in the calculation of the women’s empowerment index for Africa developed by the International Centre for Equity in Health and the DHS indicator of women empowerment [25]. The DHS collects information on women’s education, access to information, and decision-making in households allowing us to select a wide variety of variables to capture the three broad dimensions of women’s empowerment, namely, economic, socio-cultural and family/interpersonal. However, some of the questions generally used in the calculation of the empowerment index were not taken into account because they seemed too subjective, not specific to women or could be influenced by the spouse (for example, ownership of a cell phone that is related to the characteristics of the household and the acceptance of wife beating that may be biased by the presence or protection of the spouse in the context of FSSA). We thus remained with a total of 16 variables. All these variables were combined into a single index using the PCA technique and presented in Table 1.

**Table 1:** Dimensions of empowerment and their operationalization

Dimensions	DHS Variables (Births Recode file)	Justification	Operationalization
Family	Age of the respondent at 1st birth Age of the respondent at 1st sex	Age at the 1st sex and age at the 1st birth help account for the possibility that empowerment is dynamic and that life course experiences shape women’s empowerment [20].	Years Years
	Who usually decides on household purchases	How married women participate in all four of the important household decisions (their own health care, major household purchases, purchases of daily household needs and visits to her family or relatives) are important for their autonomy [40]. A woman’s ability to decide on large or small purchases affects her empowerment [35].	Husband/other alone = − 1; joint = 0; respondent alone = 1
	Decision maker for using contraception		Husband/other alone = − 1; joint = 0; respondent alone = 1
	Who usually decides on visits to family or relatives		Husband/other alone = − 1; joint = 0; respondent alone = 1
	Who usually decides on respondent’s health care		Husband/other alone = − 1; joint = 0; respondent alone = 1
	Heard of family planning on radio last few months	Resources that are considered prerequisite for the exercise of women’s choice generally include paid employment, education, and media exposure [35]. Informal channels such as the mass media (television, cinema, newspaper and so on) play an important role in bringing about greater awareness of gender issues [35].	No = 0; Yes = 1
	Heard of family planning on TV last few months		No = 0; Yes = 1
	Heard of family planning in newspaper/magazine last few months		No = 0; Yes = 1
Economic	Respondent worked in last 12 months	Women’s equal access to and control over economic resources have impact on their economic empowerment [35]. The way women’s contributions are valued and women’s ability to negotiate a fairer deal for themselves enhance their empowerment [23].	No = 0; In the past year = 1; Have a job, but on leave last 7 days = 2; Currently working = 2
	Type of earnings from respondent’s work		Not paid = 0; In-kind only = 1; Cash and in-kind = 2; Cash only = 3
	Owns a house alone or jointly		Does not own = − 1; Jointly only = 0; Alone only = 1; Both alone and jointly = 1
	Owns land alone or jointly		Does not own = − 1; Jointly only = 0; Alone only = 1; Both alone and jointly = 1
Sociocultural	Women education	A woman’s level of formal schooling and her exposure to media affect empowerment [35].	Years
	Frequency of listening to radio		Not at all = 0; Less than once a week = 1; At least once a week = 2
	Frequency of reading newspaper or magazine		Not at all = 0; Less than once a week = 1; At least once a week = 2

#### Econometric estimation

The first model analyses the association between empowerment and ideal number of children. In this regression model the dependent variable is ideal number of children for each married woman. In this situation, Poisson Regression Model and Negative Binomial Regression Model have statistical advantages over OLS [41, 42]. In this study, we used Negative Binomial Regression Model. Negative binomial regression models do not assume an equal mean and variance and particularly correct for overdispersion in the data, which is when the variance is greater than the conditional mean [43, 44].

Second, to examine the association between empowerment and a married woman’s ability to have just the desired number of children, we used multivariable logistic regression to model the probability of having more children than desired. Given the low probability of young women to have completed child-bearing, this study considers only married women aged 35 and over [12]. A second model was estimated to examine the independent contribution of the three empowerment indicators. Each indicator was included alone in the model, with control variables. This second model highlights the dimensions of empowerment that are significantly associated with women’s fertility preferences.

### Source of data

The data used in this paper are from the most recent Demographic and Health Surveys (DHS) conducted in these countries, namely, 2010 for Burkina Faso, 2014 for Chad, 2012 for Mali and Niger. DHS Program collects data that are comparable across countries. These data are nationally representative and comprise three questionnaires: a Household Questionnaire, a Women’s Questionnaire, and a Men’s questionnaire.

In order to facilitate the analysis of data, DHS has developed the concept of recode files. There are seven common types of recode data files associated with the core questionnaires. Birth Recode presents the full birth history of all women interviewed including information on pregnancy and postnatal care as well as immunization and health for children born in the last 5 years. This file can be used to generate health indicators as well as fertility and mortality rates. It contains all data needed for our study. For our estimations, we mainly used the births recodes files. The unit of analysis is the married woman aged 35 and over [9]. Given that young women of childbearing age are less likely to completed childbearing, a married woman’s ability to have just the desired number of children (number of living children minus the ideal number of children) can only be measured efficiently for women aged 35 and over.

This study therefore focuses on a more homogeneous category of women who face the same constraints in decision-making within the household. It is important to note that the term “married woman” is not limited to those whose union has been formalized through civil marriage, but also refers to all women who are traditionally married or who are living with a partner [34].

## Results

### Descriptive statistics

#### Demographic and social characteristics

Table 2 shows that on average, nearly 70% of households in each country included in the analysis had seven or more people. Less than a third of the children born alive to women aged 35 years and above in each country had died by the time of the survey (16% in Burkina Faso, 14% in Mali, 28% in Niger, and 15% in Mali). The results further show that more than 70% of the spouses of women aged 35 and above have no education (88% in Burkina Faso, 83% in Mali, 85% in Niger, and 70% in Chad). In addition, majority of women live in rural areas (79, 76, 76 and 79% in Burkina Faso, Mali, Niger and Chad, respectively).

**Table 2:** Summary statistics of explanatory variables

Variables	Burkina Faso	Mali	Niger	Chad
Social and demographic				
Place of residence (%)				
Rural	79.1	75.8	76.0	78.6
Urban	28.9	24.1	23.9	21.3
Husband education (%)				
None	88.1	82.5	86.1	75.4
Primary	8.1	6.9	7.6	13.9
Secondary	3.3	8.4	4.1	9.1
Tertiary (High school)	0.5	2.1	2.2	1.7
Household size (%)				
1–3	4.3	3.4	4.0	6.1
4–6	26.4	29.0	26.3	26.5
7–9	32.1	39.0	35.5	38.4
10 –	37.3	28.5	34.1	29.0
No. of living children (%)				
0	0.2	0.1	0.3	0.1
1–2	3.0	3.8	2.4	2.0
3–4	21.0	20.7	16.7	12.8
>=5	75.8	75.3	80.6	85.00
Religion (%)				
None	1.2	1.8	0.1	2.6
Muslim	58.0	93.0	99.1	66.8
Christian	28.5	4.1	0.5	30.2
Animist	12.2	1.1	0.2	0.3
Women				
Mean ideal number of children v613	6.9	6.7	10.2	9.5
Ideal no. of children (%)				
0–2	0.8	2.1	0.7	0.7
3–5	27.3	25.0	7.2	8.9
>= 6	71.8	72.8	92.1	90.3
Had more Children than ideal (%)	38.7	42.3	13.4	16.4
Husbands				
Husband ideal number of children	6.6	8.1	11.1	12.7
Ideal no. of children (%)				
0–2	4.0	1.4	0.6	1.4
3–5	47.6	24.8	15.9	16.1
>= 6	48.5	73.8	83.5	82.5
Ideal no. of children relative to wife’s (%)				
Agrees	28.1	19.3	9.1	13.3
Higher	55.1	34.8	44.2	34.1
Lower	3.2	4.2	1.3	2.5
Number of women	4020	1307	1160	837

#### Fertility preferences

The average ideal number of children per woman is 6.9, 6.7, 10.2 and 9.5 in Burkina Faso, Mali, Niger and Chad, respectively (Table 2). In the case of husbands, the ideal number of children is much higher: more than 50% of husbands desired a similar number of children as their wives. In addition, the highest proportion of women reported having more children than their ideal number was found in Mali (42%), followed by Burkina Faso (39%), Chad (16%) and Niger (13%).

### Empowerment and preferences regarding fertility

#### Women’s empowerment index

Because the scores are standardized, negative values imply lack of empowerment while positive values indicate some degree of empowerment. Countries with a positive empowerment index are the best positioned in terms of empowerment. Table 3 shows a significant difference between two groups of countries. On the one hand is the first group of countries (Burkina Faso and Chad), with a positive empowerment score for women aged 35 years or older and on the other hand, the second group (Mali and Niger) with a negative empowerment score. Increased empowerment of women in Burkina Faso and Chad can be linked to changes in national and local legislation; the role of local institutions that empower women, women’s role in key decision-making within households; recognition of the importance of the tasks usually performed, how they organize themselves to enhance their empowerment and increased employment and education. Table 3 also presents the empowerment scores by type of indicator. This categorization indicates the relative importance of each factor in the empowerment index. Two groups of countries also stand out. Mali is the best positioned in terms of socio-cultural empowerment while Chad is the best positioned in terms of economic empowerment. Likewise, Burkina Faso, Mali and Chad are the best positioned in terms of familial empowerment. Niger is the worst positioned for all dimensions. .

**Table 3:** Women’s empowerment index

Variables	Burkina Faso	Mali	Niger	Chad
Empowerment index – national	5.18 e-10(0.637)	-3.15e-09(0.616)	-2.82e-09(0.721)	5.10e-09(0.609)
Empowerment index – rural	− 0.562	− 0.565	− 0.739	− 0.62
Empowerment index – urban	1.0545	0.691	0.81	1.33
Economic Empowerment index - national	−7.53e-09(0.721)	−4.06e-09(0.954)	−1.36e-08(0.506)	5.18e-09(0.4351)
Economic Empowerment index - rural	−0.290	0.185	0.153	0.03
Economic Empowerment index - urban	0.0729	− 0.524	− 0.367	− 0.11
Socio-cultural empowerment index-national	−7.21e-09(0.952)	3.74e-09(0.3411)	−9.06e-09(0.789)	− 1.47e-08(0.4119)
Socio-cultural empowerment index-urban	0.8095	0.878	0.626	0.89
Socio-cultural empowerment index-rural	−0.2137	− 0.279	− 0.197	− 0.24
Familial empowerment index-national	2.38e−11(0.723)	8.14e-10(0.608)	-1.91e-09(0.823)	1.38e-10(0.56)
Familial empowerment index-urban	0.6389	0.088	0.56	0.74
Familial empowerment index-rural	− 0.3559	− 0.082	− 0.431	− 0.40

(): Cronbach’s alpha reliability test (Scale reliability coefficient)

5.18 e-10 means 5.18 × 10^−10^

Regardless of the country, the results show a wide disparity between urban and rural women. Women living in urban areas are much more empowered than their rural counterparts. Rural women are significantly less likely to take part in decision making than urban women [40]. Our findings corroborate those of Mahmud et al. [35] who found that social norms and intra-household gender-related constraints greatly influence women’s possibility of being empowered in rural areas. However, the results should be interpreted with caution for the reason that many cities in low-income countries are characterized by informal settlements/slums, which are sometimes characterized by worse health indicators than rural areas.

#### Empowerment and ideal number of children

Results of the Negative Binomial Regression Model are similar across countries (Table 4). Generally speaking and regardless of the country, the number of children considered ideal by women decreases as the empowerment index increases. More empowered women desire significantly fewer children compared with their less empowered counterparts. However, the association between the different dimensions of empowerment and desired number of children varies across the countries considered. For instance, economic and family factors are significant in Burkina Faso, economic and socio-cultural factors are significant in Mali, family factors are significant in Chad, while socio-cultural factors are significant in Niger. In addition, a woman’s living environment, household socio-economic status, the spouse’s level of education, and the household size were significantly associated with desired number of children. Our findings showed that the number of children considered ideal by women decreases as the spouse’s level of education increases, regardless of the country. The number of children considered ideal by women decreases as household socio-economic status increases in Burkina Faso and Chad while higher household socio-economic status in Mali and Niger increases significantly the desire for more children. Surprisingly, women from large households significantly desire more children in most of the countries considered.

**Table 4:** Coefficient estimates from negative binomial regression model examining association between women’s empowerment and ideal number of children

Variables	Burkina Faso		Mali		Niger		Chad	
	Model1	Model2	Model1	Model2	Model1	Model2	Model1	Model2
Women’s empowerment								
Empowerment index	−0.02***(− 0.03--0.01)		− 0.07**(− 0.02—0.01)		− 0.02**(− 0.03—0.00)		−0.01**(− 0.01—0.004)	
Economic Empowerment index		−0.01***(− 0.02—0.00)		− 0.02***(− 0.04—0.04)		−0.002(− 0.02–0.02)		0.04***(0.03–0.06)
Socio-cultural empowerment index		0.00(−0.01–0.01)		−0.01*(− 0.02–0.00)		−0.03***(− 0.04—0.01)		− 0.01(− 0.02–0.004)
Familial empowerment index		−0.03***(− 0.04—0.02)		−0.004(− 0.02–0.01)		0.00(− 0.01–0.01)		−0.02**(− 0.04--0.001)
Controls variables								
Place of residence								
Rural	0.10(−0.01–0.04)	0.02(− 0.01–0.05)	0.04(− 0.01–0.10)	0.04(− 0.02–0.10)	0.14***(0.08–0.19)	0.15***(0.10–0.21)	−0.31(− 0.38—0.24)	−0.33(− 0.40—0.26)
Household wealth index								
Poor	−0.08***(− 0.12—0.05)	−0.08***(− 0.12—0.05)	0.25***(0.16–0.35)	0.25***(0.15–0.34)	0.14**(0.03–0.25)	0.15***(0.04–0.26)	−0.10***(− 0.17–0.04)	−0.09***(− 0.15—0.20)
Middle	− 0.10***(− 0.14—0.06)	−0.10***(− 0.14—0.07)	0.001(− 0.12–0.12)	0.00(− 0.12–0.12)	0.01(−0.09–0.11)	0.01(− 0.08–0.11)	−0.09***(− 0.16—0.03)	−0.08**(− 0.14—0.01)
Rich	− 0.07***(− 0.11—0.04)	−0.08***(− 0.11—0.04)	0.13***(0.05–0.22)	0.11***(0.03–0.20)	0.19***(0.09–0.28)	0.19***(0.09–0.29)	−0.10***(− 0.16—0.03)	−0.09***(− 0.16—0.03)
Richest	− 0.21***(− 0.26—0.17)	−0.22***(− 0.26—0.17)	0.01(− 0.09–0.11)	−0.01(− 0.11–0.09)	0.13**(0.03–0.23)	0.14***(0.04–0.24)	−0.47***(− 0.56—0.38)	−0.45***(− 0.54—0.36)
Husband education								
Primary	−0.02*(− 0.05–0.00)	−0.02(− 0.05–0.01)	0.03(− 0.05–0.10)	0.02(− 0.05–0.09)	0.06**(0.01–0.12)	0.07*(0.02–0.13)	− 0.03(− 0.08–0.02)	− 0.02(− 0.07–0.03)
Secondary	−0.19***(− 0.23—0.14)	−0.21***(− 0.25—0.16)	−0.08**(− 0.14—0.01)	−0.07**(− 0.14—0.01)	−0.24***(− 0.33—0.15)	−0.23***(− 0.32—0.14)	−0.14***(− 0.20—0.07)	−0.12***(− 0.18—0.06)
Tertiary (High school)	− 0.16***(− 0.27—0.06)	−0.20***(− 0.31—0.10)	−0.15***(− 0.26—0.04)	−0.13**(− 0.24—0.02)	−0.50***(− 0.61—0.39)	−0.44***(− 0.55—0.32)	−0.12***(− 0.20—0.05)	−0.11***(− 0.18—0.05)
Household size								
4–6	−0.09**(− 0.17—0.02)	−0.10***(− 0.17—0.025)	0.22***(0.07–0.37)	0.25***(0.11–0.39)	0.22*(− 0.03–0.47)	0.21*(− 0.04–0.47)	0.09(−0.07–0.26)	0.14*(− 0.02–0.30)
7–9	− 0.03(− 0.10–0.04)	− 0.04(− 0.11–0.03)	0.30***(0.15–0.44)	0.34***(0.19–0.48)	0.29**(0.04–0.54)	0.28**(0.02–0.53)	0.25***(0.09–0.42)	0.31***(0.15–0.47)
10 and more	−0.01(− 0.08–0.06)	−0.02(− 0.10–0.05)	0.31***(0.16–0.46)	0.34***(0.20–0.48)	0.28**(0.03–0.54)	0.28**(0.03–0.53)	0.28***(0.12–0.44)	0.33***(0.17–0.48)
Polygamous union								
Yes	−0.002(− 0.03–0.02)	0.004(− 0.02–0.03)	−0.16(− 0.23—0.09	−0.16(− 0.23—0.09)	0.002(− 0.05–0.05)	−0.005(− 0.06–0.04)	−0.001(− 0.07–0.06)	0.03(− 0.05–0.10)
Religion								
Muslim	−0.31***(− 0.36—0.26)	− 0.34***(− 0.39—0.28)	−0.08(− 0.31–0.14)	−0.07(− 0.28–0.14)	−0.55(− 0.7—0.13)	−0.09*(− 0.1–0.04)	0.56(0.18–0.95)	0.51(0.13–088)
Christian	−0.36***(− 0.41—0.31)	−0.37***(− 0.42—0.32)	0.09(− 0.18–0.36)	0.09(− 0.16–0.35)	−0.1(− 0.5–0.1)	− 1.01***(− 1.2—0.8)	0.07(0.02–0.11)	0.06(0.01–0.10)
Animist/traditional	−0.31(− 0.37—0.25)	−0.33***(− 0.39—0.27)	−2.54***(− 2.1—2.4)	−1.5***(− 2.9—2.2)	−0.84(− 1.32—0.3)	−0.72***(− 1.1—0.45)	0.60**(0.09–1.02)	0.11(− 0.02–0.18)
Number of women	4020	4020	1307	1307	1160	1160	837	837

*** Significant at 1%; ** significant at 5%; significant at 10%

(): confidence intervals

Note: Model1: explanatory variables (empowerment and controls variables)

Model2: explanatory variables (desegregated empowerment index and controls variables)

#### Empowerment and ability to have the desired number of children

Results from logistic regression models examining the association between women’s empowerment and ability to achieve the desired number of children are presented in Table 5. Overall, there was no significant association between women’s empowerment and ability to have the desired number of children in Mali and Niger. However, with regard to the dimensions of empowerment, the results show that the factors that contribute positively to a woman’s ability to have the desired number of children are family factors in Burkina Faso and Chad, and economic and family factors in Mali. Table 5 shows that a woman’s living environment, household socio-economic status, the husband’s level of education and the household size significantly influence her ability to have the desired number of children. Our findings showed that rural environment influences negatively women’s ability to have the desired number of children in Niger while it is not statistically significant in the other countries included in the study. A woman’s ability to have the desired number of children increase as household socio-economic status and husband’s level of education increase. In addition, women from large households are more likely to have the desired number of children.

**Table 5:** Coefficient estimates from logistic regression analysis examining the relationship between women’s empowerment and ability to achieve fertility goals

Variables	Burkina Faso		Mali		Niger		Chad	
	Model1	Model2	Model1	Model2	Model1	Model2	Model1	Model2
Women’s empowerment								
Empowerment index	0.01*(0.007–0.04)		−0.12(−0.20—0.04)		0.12(0.023–0.22)		0.15***(0.05–0.25)	
Economic Empowerment index		0.03(−0.03–0.09)		0.24***(014–0.35)		0.11(−0.04–0.27)		−0.52***(− 0.71—0.33)
Socio-cultural empowerment index		−0.07***(− 0.12—0.03)		−0.06*(− 0.13–0004)		0.05(− 0.03–0.14)		−0.14***(− 0.23—0.04)
Familial empowerment index		0.16***(0.1–0.22)		0.14***(0.06–0.22)		0.05(−0.04–0.15)		0.23***(0.08–0.38)
Controls variables								
Place of residence								
Rural	−0.01(− 0.18–0.16)	0.0004(− 0.17–0.17)	0.19(− 0.12–0.51)	0.24(−0.09–0.57)	−1.29***(−1.80—0.78)	−1.43***(− 1.97—0.89)	13.90(13.14–14.67)	15.32(14.58–16.06)
Household wealth index								
Poor	0.01(− 0.21–0.24)	−0.01(− 0.21–0.24)	−0.94***(− 1.47—0.42)	−0.90***(− 1.4—0.37)	−1.11***(− 1.88—0.34)	−1.24***(−2.03—0.45)	0.07(− 0.55–0.69)	− 0.09(− 0.74–0.55)
Middle	0.18(− 0.04–0.41)	0.18(− 0.04–0.4)	0.06(− 0.56–0.69)	0.08(− 0.54–0.71)	−0.64**(− 1.26—0.02)	−0.72*(− 1.35—0.09)	0.82***(0.21–1.44)	0.69**(0.04–0.55)
Rich	0.04(− 0.17–0.26)	0.03(− 0.18–0.24)	0.11(− 0.37–0.60)	0.24(− 0.25–0.73)	−2.16(− 2.86—1.43)	−2.27***(− 2.99—1.55)	0.42(−0.19–1.02)	0.45(− 0.20–1.11)
Richest	0.41***(0.14–0.7)	0.33**(0.06–0.6)	0.72**(0.17–1.28)	0.92***(0.35–1.48)	−2.16(− 2.89—1.43)	−2.25(− 2.99—1.51)	14.85***(13.94–15.76)	15.95***(14.98–16.93)
Husband education								
Primary	− 0.09(− 0.27–0.11)	−0.09(− 0.3–0.09)	−0.06(− 0.50–0.37)	−0.15(− 0.57–0.27)	−1.87***(− 2.66—1.09)	−1.81***	−0.17(− 0.61–0.26)	−0.39*
Secondary	0.33**(0.04–0.61)	0.37**(0.07–0.66)	0.18(−0.15–0.53)	0.19(− 0.14–0.53)	−0.35(− 0.97–0.26)	−0.76**	0.56***(0.14–0.98)	0.17
Tertiary (High school)	−0.06(− 0.71–0.58)	0.07(− 0.61–0.77)	0.13(− 0.37–0.65)	−0.02(− 0.53–0.47)	1.02***(0.45–1.58)	1.01**	0.32(− 0.44–1.08)	0.14
Household size								
4–6	0.88***(0.4–1.29)	0.93***(0.47–1.39)	0.19(− 0.85–1.23)	0.05(− 0.91–1.02)	0.48(0.99–1.96)	0.57(−0.94–2.08)	0.002(− 0.87–0.87)	−0.23(− 0.67–0.21)
7–9	0.85***(0.4–1.32)	0.98***(0.52–1.43)	0.52(− 0.51–1.54)	0.44(−0.52–1.41)	0.64(− 0.83–2.13)	0.70(− 0.80–2.21)	−0.45(− 1.34–0.44)	0.63***(0.19–1.07)
10 –	0.92***(0.4–1.37)	1.04***(0.58–1.50)	0.89(− 0.13–1.91)	0.87(− 0.09–1.84)	1.26(− 0.21–2.74)	1.34(−0.16–2.85)	−0.43(− 1.29–0.43)	0.24(− 0.48–0.97)
Polygamous union								
Yes	− 0.35(− 0.54—0.15)	−0.40(− 0.60—0.20)	−0.02(− 0.41–0.38)	−0.08(− 0.48–0.31)	0.36(0.25–1.58)	0.34(0.14–1.12)	−0.31(− 0.89–0.27)	−0.88(− 1.56—0.19)
Religion								
Muslim	0.89**(0.05–1.73)	1.02**(0.15–1.89)	0.43(−0.31–1.17)	0.39(− 0.33–1.12)	0.09(− 0.31–0.63)	−0.75(− 1.22—0.27)	−0.12(− 0.48–0.22)	0.04(− 0.15–0.24)
Christian	1.18***(0.34–2.02)	1.26***(0.39–2.12)	0.91**(0.17–1.75	0.66**(0.12–1.95	0.72**(0.15–1.28)	0.05(− 0.44–0.51)	− 0.405(− 0.55–0.62)	− 0.16(− 0.52–0.19)
Animist/traditional	0.99**(0.12–1.85)	1.08**(0.19–1.97)	0.97**(0.11–1.12)	− 0.92(− 2.18–0.34)	0.15(0.08–0.20)	9.04***(0.58–1.50)	−1.29(− 1.80—0.78)	0.21(− 0.07–0.37
Number of women	4020	4020	1307	1307	1160	1160	837	837

*** Significant at 1%; ** significant at 5%; significant at 10%

(): confidence intervals

Note: Model1: explanatory variables (empowerment and controls variables)

Model2: explanatory variables (desegregated empowerment index and controls variables)

## Discussion

According to our results, women’s empowerment is significantly associated with a desire for fewer children in FSSA countries studied. Our findings showed that the number of children considered ideal by women decreases as the empowerment index increases, regardless of the country. These findings are consistent with most studies which found an inverse relationship between the number of desired children and empowerment [45]. The policy implication for FSSA countries is that greater gains can be made in fertility policies, by improving women’s social status, especially in rural areas. The challenge is to identify pathways in which women’s empowerment affects their desire for fewer children in FSSA countries. In this regard, the results show key indicators: the desired number of children for women in Burkina Faso and Mali decreases with their level of economic empowerment; in Burkina Faso and Chad, it decreases with familial empowerment, and in Mali and Niger it decreases with socio-cultural empowerment.

Our result suggests that the first step to having fewer children is formulating programs to improve economic empowerment of women. The findings are consistent with those of other studies in Sub-Saharan Africa which showed that women who are empowered economically, are more likely to have positive fertility behavior [46]. Economic empowerment is based on women’s socio-economic characteristics such as participation in the labor force, having an income, and access to resources that determine their ability to improve their power relations with men. Having paid employment brings more economic independence to women, enhances their decision-making power, and encourages their access to healthcare and contraception [47]. Access to resources and credit, as well as the position of women in productive sectors are factors which, if improved upon, could significantly reduce the number of children desired by women in Burkina Faso and Mali. Policy makers should consider empowerment programs that enhance women’s access to and control over resources in order to reduce their dependence on men [48]. In the socio-cultural context of high fertility, economic independence should give women the freedom to make decisions that affect their own lives, including decisions about their fertility, such as contraceptive use and the number of children they wish to have [47, 49].

However, it is counter-intuitive that in Chad, women who are empowered economically are more likely to have more children. We hypothesized that in Chad, if parents view children as more likely to take paid work or earn market wages, they may be more inclined to have more children. Child labour remains a problem in Chad [50]. In 2017, approximately 53% of children in Chad were engaged in child labor [50]. Despite the economic empowerment of women, the existence of child labour can explain the desire for many children [50]. Child labor also can explain the fact that an improvement of a household’s standard of living increases significantly the desire for more children in Niger and Mali.

The second step to having fewer children in the countries studied is implementing policies and programs that improve women’s socio-cultural and familial empowerment. Some of the indicators of socio-cultural and familial empowerment could be the choice of the type of education that one desires to achieve for oneself or one’s children, choice of age of entry into marriage, independent choice of one’s spouse, preferred methods of contraception, and freedom to manage one’s activities. Improvement in women’s education should increase their access to modern values and ideas that promote fertility decline [47]. This finding is consistent with that of Jejeebhoy [18] and Mason [51] who found that the effect of women’s education on fertility is explained by education’s effect on empowering women. Similarly, studies show that education defines women’s empowerment in Central, Southern, and West Africa [52]. In addition, the findings of this paper show that the level of education of the spouse significantly affects the woman’s ideal number of children. Women with spouses with high levels of education desired significantly fewer children compared with those whose spouses had low education levels. One of the ways of significantly reducing fertility in FSSA would be to promote access to information through adult learning and illiteracy elimination programs. These programs should target particularly married couples in regions with the highest rates of illiteracy.

Cultural norms and values are not necessarily in tandem with reproductive success in FSSA. Generally, in Niger and Mali, religious belief system and the social structure accord both spiritual and economic rewards to high marital fertility. Changes in fertility rates, family planning and the number of children in a household reflect cultural or religious differences in most FSSA countries [53, 54]. One of the key barriers to having desired number of children is sociocultural norms especially the husband’s role as primary decision-maker and the desire for a large family. Women’s access to media could be another pathway to reduce fertility because the content may carry messages about values, which are indirectly related to fertility. The findings imply that, by taking necessary steps, mass media can be used much more adequately to reduce fertility rate in FSSA countries.

Another important finding concerns the association between women’s empowerment and their ability to have the desired number of children. In FSSA context, the results are mixed. There is a significant positive association between empowerment and the ability to have desired number of children in Burkina Faso and Chad, while there is no significant association in Mali and Niger. This suggests that greater empowerment of Burkina Faso and Chad women contributes to their ability to achieve their fertility preferences. Findings from Mali and Niger are not consistent with those of Nigeria which found significant positive associations between women’s empowerment and ability to make fertility decisions [55]. Findings further show that improvement in socio-cultural empowerment significantly reduces women’s ability to have the desired number of children in Burkina Faso, Mali, and Chad but not in Niger. These findings are unexpected and suggest that improvement of women’s ability to have the desired number of children is a big challenge which policy makers must pay careful attention to.

Interestingly, in Burkina Faso, economic empowerment is significantly associated with desired number of children but not with the ability to achieve the same. Ideal number of children and ability to limit fertility to that ideal number are two concepts that should be analyzed separately. The key pathways through which empowerment influences ideal number of children and ability to limit fertility to the desired level are not the same. In FSSA context, except for Niger, improving women’s familial empowerment affect positively their ability to have the desired number of children. Our results suggest that familial empowerment matters more than other dimensions in increasing women’s ability to make fertility decisions. These results corroborate those of previous studies which found that freedom of movement and decision-making power were positively correlated with women’s ability to make fertility decisions [55]. Upadhyay and Karasek [9] found that in Mali, negative attitudes toward wife beating were associated with women’s ability to achieve desired family size. The findings suggest that higher age at marriage for women, self-choice of mate by women and decision-making power have positive effect on their ability to achieve desired family size.

We also found that a woman’s living environment, household socio-economic status, and household size significantly influence her ideal number of children and her ability to have the desired number of children. It is counter-intuitive that women from large households significantly desire more children in most of the countries considered. A possible explanation for this might be that, having many children in these countries is still regarded as the safest pension, the best insurance policy [56]. Our findings are consistent with those of previous studies which showed that in Mali, women with many children enjoy more rights and authority within households [45, 57] and hold a much more significant social position [58]. It is possible that the society gives a higher social rank, greater privileges and greater authority to families with many children [59]. According to O’Regan and Thompson [59], in societies where ideal family size is high and male spousal reproductive preferences are honored, social norms may limit women’s choices around family planning and use of modern contraceptives.

### Limitations of the study

The findings of the paper may be influenced by certain limitations. One of them is that individual, family and community factors beyond those used to compute the empowerment index can have a greater influence on a woman’s ability to have the desired number of children. These include people’s perception of abortion, misinformation about fertility, social relations, spatial mobility and traditional ideologies. In addition, the association between women’s empowerment and fertility preferences could be linked to changes in national and local legislation, which are equally important factors. Another limitation is that due to the cross-sectional nature of the data used, we can only examine the association between women’s empowerment and fertility outcomes but cannot establish causal effects. In addition, literature suggests that there could be a reverse relationship between the two indicators whereby fertility preferences are hypothesized as a cause of women’s empowerment. Such reverse relationship was not examined in the present paper. Although we limited the analysis to women aged 35 years and above to account for those who had completed childbearing, there could be some women in that age group who could still give birth in the future thereby leading to bias in the measurement of ability to have the desired number of children.

## Conclusion

We found that greater empowerment among women is likely to lead to small family norms in high fertility FSSA. Family empowerment enhances women’s ability to have the desired number of children, especially with respect to decision-making within the household. Paid employment, access to and control over resources, as well as the position of women in productive sectors of the economy are factors which, if improved upon, could significantly reduce the ideal number of children. Greater gains can be made in fertility policies and programs, by improving women’s social status, especially in rural areas. In addition, by taking necessary steps, mass media can be used much more adequately to reduce the ideal number of children in FSSA. Finally, the desire for many children could also be due to their participation in income-generating activities to improve the household’s socio-economic status.

## Abbreviations

DHS: Demographic and Health Surveys
FSSA: Francophone Sub-Saharan Africa
PCA: Principal Component Analysis
SSA: Sub-Saharan Africa
SSA: Sub-Saharan Africa
